# The Role of Annealing Process in Ag-Based BaSnO_3_ Multilayer Thin Films

**DOI:** 10.1186/s11671-016-1579-2

**Published:** 2016-08-20

**Authors:** Muying Wu, Shihui Yu, Lin He, Lei Yang, Weifeng Zhang

**Affiliations:** 1 School of Electronic Engineering, Dongguan University of Technology, Guangdong Dongguan, 523808 China; 2 School of Physics and Electronics, Henan University, Kaifeng, 475004 China

**Keywords:** TCFs, BaSnO_3_, Magnetron sputtering, Multilayer, Thin films

## Abstract

The BaSnO_3_/Ag/BaSnO_3_ multilayer structure was designed and fabricated on a quartz glass by magnetron sputtering, followed by an annealing process at a temperature from 150 to 750 °C in air. In this paper, we investigated the influence of the annealing temperature on the structural, optical, and electrical properties of the multilayers and proposed the mechanisms of conduction and transmittance. The maximum value of the figure of merit of 31.8 × 10^−3^ Ω^−1^ was achieved for the BaSnO_3_/Ag/BaSnO_3_ multilayer thin films annealed at 150 °C, while the average optical transmittance in the visible ranges was >84 %, the resistivity was 5.71 × 10^−5^ Ω cm, and the sheet resistance was 5.57 Ω/sq. When annealed at below 600 °C, the values of resistivity and transmittance of the multilayers were within an acceptable range (resistivity <5.0 × 10^−4^ Ω cm, transmittance >80 %). The observed property of the multilayer film is suitable for the application of transparent conductive electrodes.

## Background

Transparent conducting thin films (TCFs) are functional materials that combine high optical transparency with low resistivity, which are in demand in a variety of promising applications including organic photovoltaic cells, gas sensors, energy–efficient windows, photocatalysts, flat panel displays (FPDs), and organic light-emitting diodes (OLEDs) [1–5]. Indium tin oxide (ITO) has been investigated extensively and applied widely [6–8]. However, indium is a very costly material for its scarcity [9, 10]. Therefore, alternative new easy–to–handle TCF materials have attracted great technological and scientific interest. Recently, a novel structure with a three–layer system of oxide/metal/oxide (O/M/O) has attracted great attention [11–15]. With this O/M/O sandwich structure, the electrical conductivity of TCFs can be significantly improved with a very thin metal film without the degradation of optical transmittance because the reflection from the metal in visible region can be suppressed by the oxide layers [16, 17]. Among metallic materials, Au, Ag, Cu, and Al have been practically used as the thin metal layer because of their very low resistivity [11, 13, 14, 16]. However, both the metal Cu and Al are sensitive to oxygen, and the heat stability is poor. In addition, the metal Au is relatively expensive. These prevent them from being the optimal choice for application. Ag is a good candidate for such a multilayer film due to its relatively low cost, good chemical stability, and thermal tolerance [17]. Therefore, we selected Ag as the metal layer in this work.

The barium stannate (BaSnO_3_, BSO) has recently attracted much attention due to the advantages in physical properties of perovskites. These perovskite barium stannate materials have great potential applications for innovative micro- and nano-electronic devices [18–20]. BSO is insulator, the band gap value is 3.23–4.02 eV and optical transmittance in the wavelength range from 380 to 2600 nm is above 90 % [19, 21]. In addition, the chemical and thermal stability under hydrogen plasma process is very good. As a result, the BSO/Ag/BSO (BAB) multilayer thin films have the advantages of low resistivity, high optical transmittance, and good chemical and thermal stability.

It is well known that many photo-electronic devices are usually prepared in an appropriate high temperature. Therefore, the thermal stability of BAB multilayer thin films has to be evaluated before its widely practical applications. In this work, a sandwich structure of BaSnO_3_/Ag/BaSnO_3_ multilayer thin film was designed and deposited onto quartz substrates by RF and DC magnetron sputtering. The influence of annealing temperature on the structural, optical, and electrical properties of the BAB multilayer films was investigated and the mechanism was proposed.

## Methods

The BAB multilayer thin films were prepared on quartz substrates by RF magnetron sputtering of a BaSnO_3_ ceramic target and DC magnetron sputtering of a Ag target (99.99 % purity, 60-mm diameter, 0.30-cm thickness) in an inline magnetron sputtering deposition system at room temperature (Model#, manufacturer of magnetron sputtering deposition system). The starting materials of BaSnO_3_ ceramic target were weighed and mixed with raw materials of BaCO_3_ (99.99 %) and SnO_2_ (99.99 %) according to the stoichiometric mole ratio of the BaSnO_3_, which were milled for 6 h and sintered at 1400 °C for 12 h to fabricate BaSnO_3_ ceramic target. The distance from the target to the substrate was fixed at 6 cm. The working pressure for deposition was maintained at 0.5 Pa in a high purity (99.999 %) Argon gas. The sputtering power of both the top and bottom BaSnO_3_ layers was 50 W, and the thickness was 50 nm. The sputtering power of Ag mid-layer was 40 W, and the thickness was 9 nm. There was no break in vacuum at any stage during the preparation of the films. After the deposition, the BAB multilayer thin films were annealed at 150, 300, 450, 600, and 750 °C for an hour in air, respectively.

The thickness was measured by Alpha–Step D-100 profilometer (KLA–Tencor, CA, USA). The crystal structure was characterized by X-ray diffraction system (XRD; DX–2500, FangYuan, PR China) equipped with a Cu–Kα radiation source (1.542 Å). The electrical properties (including electrical resistivity, Hall mobility, carrier concentration, and sheet resistance) were measured by Hall measurements in the van der Pauw configuration (Ecopia HMS 3000 Hall System, Republic of Korea) and four-point probe instrument (SX1934, SuZhou, PR China). Optical transmittance and absorption spectra were measured with a UV–VIS spectrophotometer (Varian Cary 5000, USA) in the wavelength range 300–800 nm.

## Results and Discussion

**Fig. 1:**
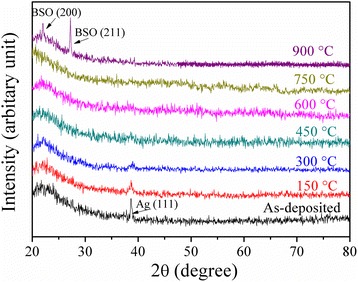
XRD patterns of the as-deposited BAB multilayer thin films before and after annealing

In multilayer thin films, the optical and electrical properties strongly depend on the microstructure of the films. The X-ray diffraction measurements were used to detect the microstructure of BAB multilayer thin films. Figure 1 shows the XRD patterns of the as-deposited BAB multilayer thin films before and after annealing, respectively. All the multilayer thin films except the films annealed at 900 °C do not show any characteristic X-ray diffraction peaks of BSO, due to the crystallization temperature of BSO at above 800 °C. (1 1 1) peak at around 2*θ* = 38.5° are observed from the Ag in the BAB multilayer thin films. The intensity of (1 1 1) peak is weakened when the annealing temperature is increased, which can be understood as the fact that the Ag mid-layer becomes discontinuous or isolated islands due to the diffusion and oxidation of numerous Ag atoms, leading to destruction of Ag layer. After annealed at 750 °C, the (1 1 1) peak disappears, indicating the Ag mid-layer do not exist. After annealed at 900 °C, the X-ray diffraction pattern from BAB multilayer thin films exhibits multiple crystalline structures with (200) and (211). The disappearance of Ag peaks may be due to the Ag atoms are incorporated into the interstitial lattice sites of BSO at high temperature.

**Fig. 2:**
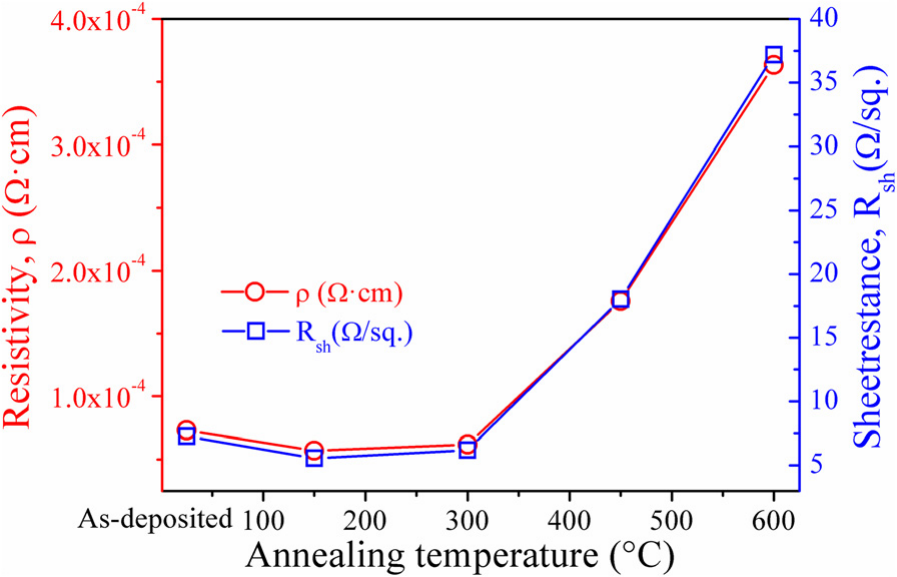
Electrical resistivity and sheet resistance of the BAB multilayer thin films as a function of the annealing temperature

Figure 2 shows the variation in electrical resistivity and sheet resistance of the BAB multilayer thin films as a function of the annealing temperature. The resistivity of as-deposited BAB multilayer thin films is 7.30 × 10^−5^ Ω cm at room temperature. With the increase in annealing temperature, the resistivity initially decreases to a minimum value (*ρ* ∼ 5.71 × 10^−5^ Ω cm) at 150 °C, and then increases gradually to 3.64 × 10^−4^ Ω cm at 600 °C. However, due to the further increase in the annealing temperature to 750 °C, the multilayer thin films become an insulator. As shown in Fig. 2, the variation of the sheet resistance upon the temperature is similar to that of the resistivity, which is due to the fact that the sheet resistance approximately equals to the ratio of the resistivity to the thickness of the films.

**Fig. 3:**
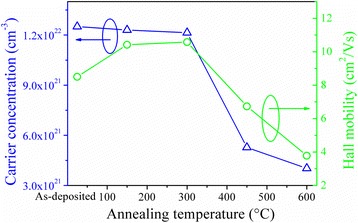
Carrier concentration and mobility of BAB multilayer thin films annealed at different temperatures

The change in resistivity and sheet resistance can be ascribed to the changes in carrier concentration and mobility. In order to attain the detailed variation information in resistivity of the BS/Ag/BS multilayer thin films as the function of the annealing temperature, the resistivity can be explained by the following basic relation: [22]

1 $$ \rho =\frac{1}{ne\mu } $$

where *ρ* is resistivity, *n* is the carrier concentration, *e* is the charge of electron, and *μ* is the carrier mobility. Figure 3 shows the carrier concentration and mobility of BAB multilayer thin films annealed at different temperatures. The single BaSnO_3_ film is an insulator [20]. Upon insertion of the 9-nm thick Ag layer, the carrier concentration increases sharply to 1.25 × 10^22^ cm^−3^. The positions of Fermi levels of the Ag relative to the vacuum level are different from BSO. The work function of BSO is higher than that of Ag [23]; thus, an Ohmic contact is formed at the interface between the metal Ag and the BSO. When the Ag layer contacts with BSO layers, there is significant injection of electrons into the BS layer due to the accumulation of electrons occurs in the BSO layers. The conduction and valence bands of BSO curve downward due to these electrons transfer, until receiving a thermodynamic equilibrium, since the Fermi level is constant throughout the structure in thermodynamic equilibrium. As a consequence, the electrons in the Ag mid-layer will flow into BSO layers without existence of barrier and high carrier concentration is detected in the BAB multilayer thin films. The carrier concentration of BAB multilayer thin films gradually decreases with the increase in the annealing temperature to 600 °C. Because the Ag atoms have a high diffusion coefficient [24], Ag atoms have enough energy to diffuse though BSO layers, and oxygen atoms diffuse through silvers when increasing the annealing temperature, the Ag mid-layer is destructed and the Ag mid-layer becomes discontinuous or even isolated islands. Due to the further increase in the annealing temperature to 750 °C, the Ag mid-layer disappears as the result in non-conductive multilayer thin films. Similarly, the mobility initially increases, reaching a maximum value (*μ* ∼ 10.42 cm^2^/Vs) at 150 °C, decreases gradually to 3.78 cm^2^/Vs when increasing the annealing temperature. The initial increase in mobility is due to the quality of BSO layers becomes better. When the annealing temperature is above 200 °C, the decrease in the mobility with annealing temperature is related to an increase in discontinuous scattering sites due to the existence of discontinuous Cu layer or Cu islands.

**Fig. 4:**
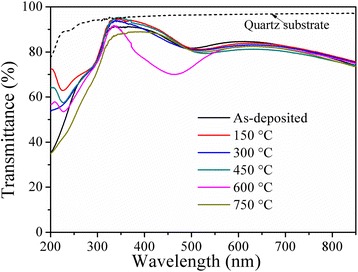
Optical transmittance of BAB multilayer thin films as a function of the annealing temperature

Figure 4 shows the optical transmittance of BAB multilayer thin films as a function of the annealing temperature. The average optical transmittance can be determined as follows [25]:

2 $$ {T}_{\mathrm{av}}=\frac{{\displaystyle \int V\left(\lambda \right)}T\left(\lambda \right)d\lambda }{{\displaystyle \int V\left(\lambda \right)d\lambda }} $$

where *T*_av_ is the average optical transmittance in the visible range (380–780 nm), *V(λ)* is the luminous spectral efficiency function defining the standard observer for photometry [26], and *T(λ)* is the measured transmittance of BAB multilayer thin films. According to Eq. (2), the average transmittance of quartz substrate is above 95 %, the average transmittances of BAB multilayer thin films annealed at different temperature can be calculated as 83.9, 84.1, 83.0, 82.3, 78.3, and 82.5 %, respectively. After annealed at 150 °C, the BAS multilayer thin films showed a maximum average transmittance of 84.1 %. The average transmittance gradually decreased as the annealing temperature further increased to 600 °C. As we all know that the BSO layers can decrease the reflectance from the surfaces of Ag and substrate and promote high optical transmission in the visible region. However, the antireflection effect of the BSO layers is weakened due to the diffusion of Ag atoms into the BSO layers at a high annealing temperature, and a great amount of lights are scattered by the surfaces of Ag and substrate. Therefore, the average transmittance decreased. In addition, the light scattering loss caused by the discontinuous Ag layer or isolated islands can also be a possible reason for the relatively low transmittance at high temperature. As the annealing temperature further increased to 750 °C, the average transmittance increased again, which can be ascribed to the disappear of Ag mid-layer.

**Fig. 5:**
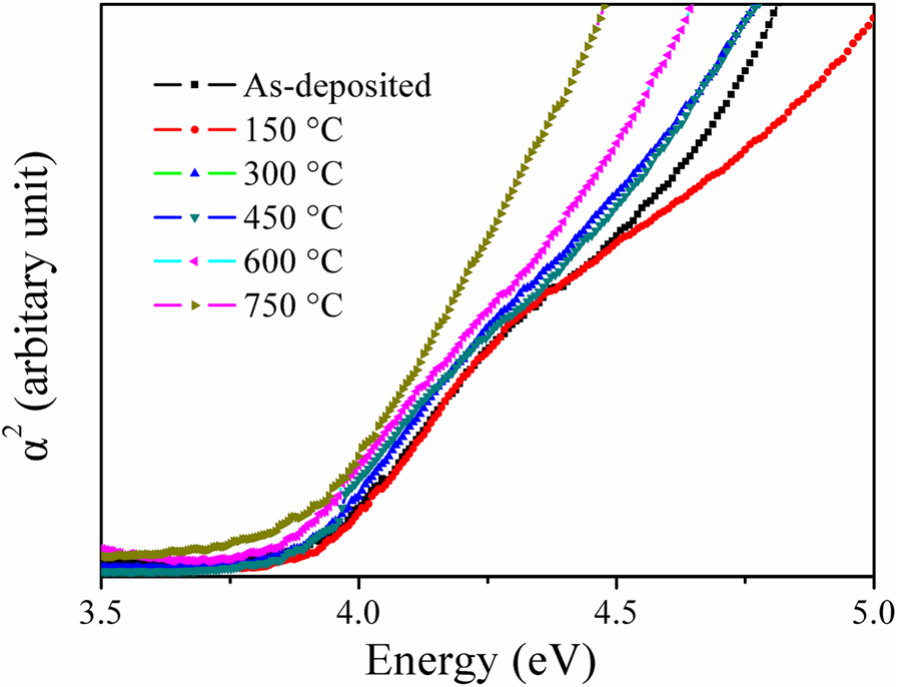
Optical absorption coefficient (*α*) vs. photon energy (hv) for the BAB multilayer thin films with varying annealing temperature

Figure 5 shows the change in optical absorption coefficient (*α*) with photon energy (hv) for the BAB multilayer thin films with varying annealing temperature on quartz substrate. To determine the energy gap, Eg, the following equation is used [27–29]:

3 $$ {(ahv)}^2=C\left(hv-\mathrm{Eg}\right) $$

where *h* is the Planck constant, *α* is the frequency of incident photon, and *C* is a constant depending on the material. By extrapolating the linear region of *α*^2^ − (*hv*) plots to the energy axis, the Eg values can be obtained. From the inset, the optical band gap of the as-deposited BAB multilayer thin film is 3.91 eV after annealed in air. It can be found that the optical band gap of the multilayer thin films narrows with the increase in the annealing temperature. According to Fig. 3, we can know that the carrier concentration for multilayer thin films increases as the annealing temperature decreases. This means that the band gap energy Eg increases with the carrier concentration increasing. The Fermi energy penetrates into the conduction band of the degenerate semiconductor, due to an increase in the carrier concentration, which leads to the energy band widening. This phenomenon is usually considered as the results of the Fermi level band filling in crystals, known as the Burstein–Moss effect [30–32].

**Fig. 6:**
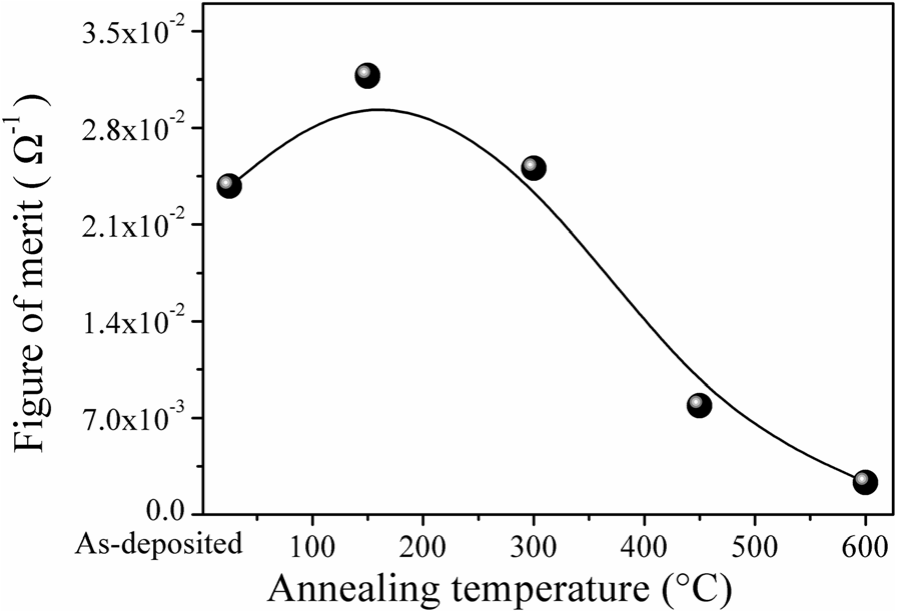
FOM values of as-deposited BAB multilayer thin films and with different annealing temperature

The figure of merit (FOM) is an important index that briefly describes the relationship between sheet resistance and optical transmittance for the TCFs. A figure of merit, as defined by Haacke [31], is commonly used to reflect the trade-off between optical transmittance and electrical conduction, which can be defined as [33]

4 $$ \mathrm{F}\mathrm{O}\mathrm{M}=\frac{{T_{\mathrm{av}}}^{10}}{R_{\mathrm{sh}}} $$

where ${T_{\mathrm{av}}}^{10}$ is the optical transmittance of multilayer thin films and *R*_sh_ is the sheet resistance. Considering the application of the multilayer thin films for display devices, the average optical transmittance in the visible range (380–780 nm) is used in Eq. (4). Figure 6 shows a plot of FOM for the as-deposited BAB multilayer thin films and with different annealing temperature. From the plot, it can be seen that the FOM value initially increased with annealing temperature, reaching a maximum value (31.8 × 10^−3^ Ω^−1^) at 150 °C, and then decreased with further increase in the annealing temperature. The largest FOM value was obtained when the multilayer thin films were annealed at 150 °C. It can be concluded that the BAB multilayer thin films annealed at 150 °C was a suitable structure for application in transparent electrode.

## Conclusions

In conclusion, the BAB multilayer structure has been designed and fabricated on quartz glass by simultaneous RF magnetron sputtering of BaSnO_3_ and DC magnetron sputtering of Ag, followed by annealing at a temperature from 150 to 750 °C in air. The influence of the annealing temperature on the structural, optical, and electrical properties of the multilayers was experimentally investigated. A good transparent conductive multilayer thin film with an average optical transmittance of 84.1 %, resistivity of 5.71 × 10^−5^ Ω cm, and the sheet resistance of 5.57 Ω/sq was achieved with an annealing temperature at 150 °C, while the figure of merit reached a maximum of 31.8 × 10^−3^ Ω^−1^, and the band gap is 3.90 eV. The values of resistivity and transmittance of the multilayers were within an acceptable range (resistivity <5.0 × 10^−4^ Ω cm, transmittance >80 %). when annealed at below 600 °C. These experimental results indicate that BAB multilayer thin films are attractive candidates for application in transparent conductive electrodes of optoelectronic devices.

## Abbreviations

BAB: Barium stannate/Ag/barium stannate
BSO: Barium stannate
FPD: Flat panel display
ITO: Indium tin oxide
O/M/O: Oxide/metal/oxide
OLED: Organic light emitting diode
TCF: Transparent conducting thin films
XRD: X-ray diffraction

## References

[1] Hagendorfer H, Lienau K, Nishiwaki S, Fella CM, Kranz L, Uhl AR, Jaeger D, Luo L, Gretener C, Buecheler S, Romanyuk YE, Tiwari AN (2014). Highly transparent and conductive ZnO: Al thin films from a low temperature aqueous solution approach. Adv Mater.

[2] Zakutayev A, Perry NH, Mason TO, Ginley DS, Lany S (2013). Non-equilibrium origin of high electrical conductivity in gallium zinc oxide thin films. Appl Phys Lett.

[3] Yu S, Li L, Zhang W, Sun Z, Zheng H (2015). Fully transparent thin-film varactors: fabrication and performance. J Mater Chem C.

[4] Ye ZY, Lu HL, Geng Y, Gu YZ, Xie ZY, Zhang Y, Sun QQ, Zhang DW (2013). Structural, electrical, and optical properties of Ti–doped ZnO films fabricated by atomic layer deposition. Nanoscale Res Lett.

[5] Yu S, Li L, Sun Z, Zheng H, Dong H, Xu D, Zhang W (2015). Characteristics of transparent conducting W–Doped SnO_2_ thin films prepared by using the magnetron sputtering method. J Am Ceram Soc.

[6] Lin HK, Cheng KC, Huang JC (2015). Effects of laser annealing parameters on optical and electrical properties of ITO/metallic glass alloy Bi–layer films. Nanoscale Res Lett.

[7] Yu S, Ding L, Zheng H, Xue C, Chen L, Zhang W (2013). Electrical and photoelectric properties of transparent Li–doped ZnO/ZnO homojunctions by pulsed laser deposition. Thin Solid Films.

[8] Wu M, Yu S, Chen G, He L, Yang L, Zhang W (2015). Structural, optical, and electrical properties of Mo–doped ZnO thin films prepared by magnetron sputtering. Appl Surf Sci.

[9] Pattini F, Annoni F, Bissoli F, Bronzoni M, Garcia JP, Gilioli E, Rampino S (2015). Comparative study about Al–doped zinc oxide thin films deposited by pulsed electron deposition and radio frequency magnetron sputtering as transparent conductive oxide for Cu (In, Ga) Se_2_–based solar cells. Thin Solid Films.

[10] Noor N, Parkin IP (2013). Enhanced transparent-conducting fluorine-doped tin oxide films formed by aerosol-assisted chemical vapor deposition. J Mater Chem C.

[11] Yu S, Li L, Lyu X, Zhang W (2016). Preparation and investigation of nano-thick FTO/Ag/FTO multilayer transparent electrodes with high figure of merit. Sci Rep.

[12] Crupi I, Boscarino S, Torrisi G, Scapellato G, Mirabella S, Piccitto G, Simone F, Terrasi A (2013). Laser irradiation of ZnO: Al/Ag/ZnO: Al multilayers for electrical isolation in thin film photovoltaics. Nanoscale Res Lett.

[13] Sahu DR, Huang JL (2007). The properties of ZnO/Cu/ZnO multilayer films before and after annealing in the different atmosphere. Thin Solid Films.

[14] Al–Kuhaili MF, Al–Maghrabi MA, Durrani SMA, Bakhtiari IA (2008). Investigation of ZnO/Al/ZnO multilayers as transparent conducting coatings. J Phys D Appl Phys.

[15] Yu S, Li L, Xu D, Dong H, Jin Y (2014). Characterization of SnO_2_/Cu/SnO_2_ multilayers for high performance transparent conducting electrodes. Thin Solid Films.

[16] Kim D (2010). Low temperature deposition of transparent conducting ITO/Au/ITO films by reactive magnetron sputtering. Appl Surf Sci.

[17] Dhar A, Alford TL (2012). Optimization of Nb_2_O_5_/Ag/Nb_2_O_5_ multilayers as transparent composite electrode on flexible substrate with high figure of merit. J Appl Phys.

[18] James KK, Krishnaprasad PS, Hasna K, Jayaraj MK (2015). Structural and optical properties of La-doped BaSnO_3_ thin films grown by PLD. J Phys Chem Solids.

[19] Liu Q, Dai J, Liu Z, Zhang X, Zhu G, Ding G (2010). Electrical and optical properties of Sb-doped BaSnO_3_ epitaxial films grown by pulsed laser deposition. J Phys D Appl Phys.

[20] Luo X, Oh YS, Sirenko A, Gao P, Tyson TA, Char K, Cheong SW (2012). High carrier mobility in transparent Ba_1–x_La_x_SnO_3_ crystals with a wide band gap. Appl Phys Lett.

[21] Mizoguchi H, Chen P, Boolchand P, Ksenofontov V, Felser C, Barnes PW, Woodward PM (2013). Electrical and optical properties of Sb-doped BaSnO_3_. Chem Mater.

[22] Djessas K, Bouchama I, Gauffier JL, Ayadi ZB (2014). Effects of indium concentration on the properties of In-doped ZnO films: Applications to silicon wafer solar cells. Thin Solid Films.

[23] Reddy CG, Manorama SV, Rao VJ, Lobo A, Kulkarni SK (1999). Noble metal additive modulation of gas sensitivity of BaSnO_3_, explained by a work function based model. Thin Solid Films.

[24] Yu S, Zhang W, Li L, Xu D, Dong H, Jin Y (2013). Transparent conductive Sb-doped SnO_2_/Ag multilayer films fabricated by magnetron sputtering for flexible electronics. Acta Mater.

[25] Driscoll WG, Vaughan W (1978). Handbook of optics.

[26] Yu S, Zhang W, Li L, Xu D, Dong H, Jin Y (2014). Optimization of SnO_2_/Ag/SnO_2_ tri-layer films as transparent composite electrode with high figure of merit. Thin Solid Films.

[27] Tauc J, Grigorovici R, Vancu A (1966). Optical properties and electronic structure of amorphous germanium. Phys Status Solidi B.

[28] Long H, Chen A, Yang G, Li Y, Lu P (2009). Third-order optical nonlinearities in anatase and rutile TiO_2_ thin films. Thin Solid Films.

[29] Marotti RE, Guerra DN, Bello C, Machado G, Dalchiele EA (2004). Bandgap energy tuning of electrochemically grown ZnO thin films by thickness and electrodeposition potential. Sol Energy Mater Sol Cells.

[30] Moss TS (1954). The interpretation of the properties of indium antimonide. Proc Phys Soc Sec B.

[31] Chen A, Long H, Li X, Li Y, Yang G, Lu P (2009). Controlled growth and characteristics of single-phase Cu_2_O and CuO films by pulsed laser deposition. Vacuum.

[32] Burstein E (1954). Anomalous optical absorption limit in InSb. Phys Rev.

[33] Haacke G (1976). New figure of merit for transparent conductors. J Appl Phys.

